# Manila Dental Society—Resolutions on the Death of Dr. Theodore Menges and Dr. George H. Cushing

**Published:** 1900-11

**Authors:** 


### Obituary.

### MANILA DENTAL SOCIETY.

### RESOLUTION ON THE DEATH OF DR. THEODORE MENGES.

THE Manila Dental Society, numbering among its members former associates and pupils of Dr. Theodore Menges, have learnt with great sorrow of his demise while yet in the prime of manhood, and at a meeting held this day have adopted the following resolution, of which a copy is ordered to be spread upon the minutes of the Society, one to be sent to his sorrowing widow and others to the dental journals of the United States:

WHEREAS, Our departed friend, by his untiring energy, by his constant devotion to duty, has done more than his share for the uplifting of dental education in the United States; and

WHEREAS, A host of his former pupils have each and all lost not merely a teacher but a true friend, adviser, and counsellor, whose constant aim was the betterment of their condition; now therefore, be it

*Resolved,* That in his death the dental profession has lost one whose efforts on her behalf have always been elevating, his former pupils have lost a worthy friend, his wife has been bereaved of a loving husband and constant companion, that with all of these we sympathize and mourn, keenly feeling, while far away from our homes and friends, that in his passing away we, too, have lost.

### RESOLUTION ON THE DEATH OF DR. GEORGE H. CUSHING.

The intelligence having been received by the members of the dental profession of Manila that the spirit of the venerable Dr. George B. Cushing has passed away, and recognizing the great loss to the dental profession by reason of his death, and furthermore feeling that the beneficent influence of his long and useful career as a practitioner and teacher are operative in even this distant land, the members of the Manila Dental Society have come together to do honor to his memory, and have adopted the following resolution:

WHEREAS, The Almighty in His infinite wisdom has spared the life of our friend and teacher until, though past the allotted life of man, he was still in possession of the strength and power of mind to be serviceable as a teacher; and

WHEREAS, The world is better by reason of his busy life, the dental profession richer in her store of knowledge, and the hearts of his friends sorrowful in the consciousness of his departure from among them; now therefore, be it

*Resolved,* That the members of this Society, by this action, add their tribute to his worth, express their sincere and heartful sympathy to his bereaved family and to the dental profession of the United States, and that this resolution be entered of record, a copy be sent to the family of the deceased and to the dental journals for publication.

LOUIS OTTOFY,
W. G. SKIDMORE,
LLOYD R. HAWLEY,
*Committee.*

MANILA, P. I., August 6, 1900.

